# Abstract Number ‐ 157: Effect of Antiplatelet Therapy in Acute Ischemic Stroke with Tandem Lesions

**DOI:** 10.1161/SVIN.03.suppl_1.157

**Published:** 2023-03-11

**Authors:** Mudassir Farooqui, Milagros Galecio‐Castillo, Afshin Divani, Amer Malik, Nils Petersen, Marc Ribo, Michael Abraham, Johanna Fifi, Waldo Guerrero, James Siegler, Thanh Nguyen, Sunil Sheth, Albert Yoo, Guillermo Linares, Nazli Janjua, Wondewossen Tekle, Marion Oliver, Syed Zaidi, Alicia Castonguay, Jessica Kobsa, Ayush Prasad, Asad Ikram, Hamza Anwer, Mary Patterson, Cynthia Zevallos, Darko Quispe‐orozco, Manual Requena, Marta Gadea, Abid Qureshi, Tiffany Barkley, Andres Dajles, Stavros Matsoukas, Ameena Rana, Mohammad Abdalkader, Sergio Marioni, Jazba Soomro, Juan Vivanco‐Suarez, Charoskhon Turabova, Randall Edgell, Maxim Mokin, Dileep Yavagal, Osama Zaidat, Mouhammad Jumaa, Ameer Hassan, Santiago Ortega‐Gutierrez

**Affiliations:** ^1^ University of Iowa Iowa City Iowa United States of America; ^2^ University of New Mexico Albuquerque New Mexico United States of America; ^3^ University of Miami Miami Florida United States of America; ^4^ Yale New Haven Connecticut United States of America; ^5^ Vall De Hebron Barcelona Spain; ^6^ University of Kansas Medical Center Kansas City Missouri United States of America; ^7^ Mount Sinai New York City New York United States of America; ^8^ University of South Florida Miami Florida United States of America; ^9^ Cooper University Hospital New Jersey United States of America; ^10^ Medical Center Boston Massachusetts United States of America; ^11^ University of Texas Health Sciences Center Houston United States of America; ^12^ Texas Stroke Center Dallas United States of America; ^13^ Saint Louis University St Louis United States of America; ^14^ Pomona Valley Hospital Medical Center Pomona United States of America; ^15^ Valley Baptist Medical Center Harlingen United States of America; ^16^ University of Toledo Toledo United States of America; ^17^ Mercy Health St. Vincent Hospital Toledo United States of America; ^18^ Texas Stroke Center Dallas United States of America; ^19^ Mercy Health St. Vincent Hospital Toledo United States of America

## Abstract

**Introduction:**

Recent studies have shown beneficial effects of Carotid artery stenting (CAS) in acute ischemic stroke patients with tandem lesions (TL). However, stent placement requires the use of antiplatelet medications to prevent in‐stent thrombosis and re‐occlusion of the artery. This must be balanced with the risk of intracerebral hemorrhage. In this multicenter study, we aimed to investigate the safety and feasibility of using antiplatelet regimens in patients with anterior circulation stroke with TLs.

**Methods:**

Patient level data were pooled from 17 centers and included patients with intracranial occlusion of ICA or M1/M2 segment of MCA with a concomitant extracranial ICA occlusion or stenosis ≥ 50%. Inclusion criteria were; age ≥ 18 years, EVT for intracranial occlusion, and underwent treatment for extracranial ICA lesions demonstrated on CTA and/or DSA. Patients were divided into groups according to the number of antiplatelets administered at the time of endovascular therapy (EVT) procedure into four groups including; 1) no antiplatelets, 2) single oral antiplatelet, 3) dual antiplatelets, and 4) intravenous antiplatelets (in combination of single or dual antiplatelets). Multivariable logistic regression models with multiple imputations were built to assess the association of primary outcome; symptomatic intracranial hemorrhage (sICH), and secondary outcomes including; modified Rankin Score (mRS) 0–2 at 90 days, and successful reperfusion (mTICI score ≥ 2b).

**Results:**

A total of 682 patients were included. Of these, 138 (20.2%) did not receive any antiplatelet therapy, while 143 (20.97%) were treated with single oral, 207 (30.35%) with dual oral, and 194 (28.5%) with intravenous combined with single or dual antiplatelets. The rate of favorable outcome was non‐significantly higher in the dual (53%) and IV‐combination (54.4%) antiplatelets, as compared with single (38.9%) and without antiplatelet (38.6%) medications. In the multivariable model, after adjusting, there was no significant differences in the sICH (single: aOR: 1.07, CI: 0.62‐1.84, p = 0.8, dual: aOR: 1.18, CI: 0.70‐1.99, p = 0.55, IV‐combination: aOR: 1.01, CI: 0.57‐1.78, p = 0.98) and functional outcome at 90 days (single: aOR: 1.15, CI: 0.63‐2.12, p = 0.64, dual: aOR: 1.03, CI: 0.55‐1.9, p = 0.9, IV‐combination: aOR: 0.83, CI: 0.42‐1.64, p = 0.59) among the study groups. Interestingly, successful reperfusion (mTICI score ≥ 2b) was significantly higher in dual oral and IV‐combination antiplatelets (single: aOR: 1.14, CI: 0.61‐2.14, p = 0.69, dual: aOR: 4.82, CI: 2.23‐10.42, p = < 0.001, IV‐combination: aOR: 3.65, CI: 1.71‐7.79, p = 0.001).

**Conclusions:**

Administration of antiplatelet medications during EVT was associated with successful reperfusion without increasing the rate of symptomatic hemorrhage in patients with anterior circulation LVO with TLs. Further large‐scale randomized studies are warranted to validate the optimal antiplatelet regimens during acute carotid artery stenting in patients with TLs.

